# Efficient embedded sleep wake classification for open-source actigraphy

**DOI:** 10.1038/s41598-020-79294-y

**Published:** 2021-01-11

**Authors:** Tommaso Banfi, Nicolò Valigi, Marco di Galante, Paola d’Ascanio, Gastone Ciuti, Ugo Faraguna

**Affiliations:** 1 The BioRobotics Institute, Scuola Superiore Sant’Anna, Pisa, Italy; 2 Department of Excellence in Robotics & AI, Scuola Superiore Sant’Anna, Pisa, Italy; 3 sleepActa S.R.L, Pontedera, Italy; 4 Department of Developmental Neuroscience, IRCCS Stella Maris, Pisa, Italy; 5 Department of Translational Research and of New Medical and Surgical Technologies, University of Pisa, Pisa, Italy

**Keywords:** Biomedical engineering, Sleep disorders, Physiology, Neurophysiology

## Abstract

This study presents a thorough analysis of sleep/wake detection algorithms for efficient on-device sleep tracking using wearable accelerometric devices. It develops a novel end-to-end algorithm using convolutional neural network applied to raw accelerometric signals recorded by an open-source wrist-worn actigraph. The aim of the study is to develop an automatic classifier that: (1) is highly generalizable to heterogenous subjects, (2) would not require manual features’ extraction, (3) is computationally lightweight, embeddable on a sleep tracking device, and (4) is suitable for a wide assortment of actigraphs. Hereby, authors analyze sleep parameters, such as total sleep time, waking after sleep onset and sleep efficiency, by comparing the outcomes of the proposed algorithm to the gold standard polysomnographic concurrent recordings. The relatively substantial agreement (Cohen’s kappa coefficient, median, equal to 0.78 ± 0.07) and the low-computational cost (2727 floating-point operations) make this solution suitable for an on-board sleep-detection approach.

## Introduction

Reliably studying human sleep in naturalistic conditions, while using non-invasive techniques, is still an unsolved problem. Currently, the gold standard to objectively study human sleep is the overnight polysomnography (PSG). In order to meet the requirements defined by the American Academy of Sleep Medicine (AASM), a PSG should simultaneously record various electrophysiological signals, i.e. electroencephalogram, electrocardiogram, electrooculogram, and electromyogram^1^. These signals are used to manually perform the so-called sleep staging. During this procedure, an expert and trained technician examines and tags the PSG recording by eye. For the entire duration of the recording, the operator is tasked with labelling the behavioral state corresponding to each non-overlapping 30 s epoch in the recording. AASM defines standard criteria for the five different behavioral states that can be assigned to each epoch: (1) waking, (2) NREM sleep N1, (3) NREM sleep N2, (4) NREM sleep N3, and (5) REM sleep. This manual approach is rather time consuming and is affected by a modest test–retest reliability: agreement between independent scorers on the same data was measured to be around 78.1 ± 9.7%^2,3^.

The administration of a PSG exam usually requires subjects to spend a night in a specialized laboratory in an unfamiliar environment, thus changing the very same sleep features, object of the study^4^. This limitation may be mitigated by using a portable PSG system, hence allowing subjects to leave the sleep laboratory and enabling the collection of data remotely in a naturalistic environment. Moreover, recording the physiological signals requires the application of several skin electrodes (around 30) and additional instrumentation potentially interfering with normal sleeping condition. While PSG remains the gold standard for its ability to directly record brain electrical activity and polygraphic signals, its application to long-term monitoring is severely limited by its invasiveness. Thus, for some common sleep disorders, such as insomnia—and particularly chronic insomnia—PSG cannot be considered the gold standard as the time window monitored by the technique is too short and the discomfort of the approach can impair sleep quality by itself.

A less invasive, clinically validated approach, is represented by Actigraphy (ACT). ACT is defined in the PubMed MESH dictionary as “*the measurement and recording of motor activity to assess rest/activity cycles*”. This technique is used in clinical and research studies where PSG is difficult to administer. ACT can be successfully used to monitor sleep longitudinally, non-invasively and in unstructured setting outside the laboratory^5–7^. Practice parameters and clinical guidelines set the perimeter of FDA-cleared ACT as an acceptably accurate estimate of sleep patterns in normal and healthy adult populations^7^. More recently, the AASM task force of sleep medicine clinicians with expertise in the use of actigraphy, provided guides and recommendation statements for clinicians using actigraphy in evaluating patients with sleep disorders and circadian rhythm sleep–wake disorders^8^. In numerous of such disorders, ranging from insomnia to central hypersomnolence, the recommendation fell into the “*conditional*” strength category, according to the GRADE process^9^. The *“conditional”* recommendation (*e.g. “We suggest…”*) versus the *“strong”* recommendation (*e.g.* *“We recommend…”*) reflects a lower degree of certainty regarding the outcome and appropriateness of the patient-care strategy for all patients. In the specific case of the application of ACT to sleep and circadian disorders, the overall quality of evidence was moderate due to imprecision. The degree of imprecision is variable according to the different ways accelerometric raw data are processed and by the mathematical models used to estimate sleep versus waking. This imprecision variability is summarized in Supplementary Table 1, displaying an overview of the performances reported by other studies using ACT in the binary sleep/wake classification concordance.

The state of the art in the field of sleep/wake classification using actigraphy data include a variety of methods to address this task. The average epoch-by-epoch accuracy of traditional algorithms with PSG scoring is 75.6 ± 3.9% (considering binary sleep/wake classification), with an associated average error of 61.3 ± 10.6 on WASO and 18.6 ± 5.1% on SE% estimation^10^. Many of these traditional algorithms do not exploit recent advances in classification techniques to achieve their task, and were proved to be inferior to machine-deep learning approaches in both epoch-by-epoch comparison and in the estimation of sleep quality and quantity metrics. In fact, deep learning algorithms scored an average accuracy of 87.7 ± 2.3%, an average WASO error 44.4 ± 2.2, and an average SE% error of 10.6 ± 0.7. To the best of our knowledge, no solution reported in the current state of the art developed models that are optimized to exploit the advances in computing and sensing hardware, nowadays commonly embedded in a variety of wearable devices. A drawback of ACT is its relatively low ability to distinguish between quiet wakefulness and sleep^11^. This feature impairs the ability of actigraphy to detect sleep onset, which is usually affected by a non-random anticipation of detection, when compared to PSG detected onset^7^. Another source of imprecision is the widespread use of brand-specific pre-processing techniques and coding of motion data derived from raw acceleration using mathematical methods (*e.g.* integration over a fixed window of time). This practice limits cross-study reproducibility of results, encumbers the pooling of datasets, and might lead to a reduction in the overall accuracy. However, the current technology allows recording of raw triaxial acceleration at both high frequency (~ 100 Hz) and resolution (~ 10bit or higher), thus promising to alleviate some of these challenges. While much of the existing research studies focused on developing algorithms for offline use, limited effort has been devoted to the possibility to embed sleep–wake classifiers on wearable devices. This approach has obvious privacy benefits as all the information processing takes place locally and offline without the need to rely on remote servers. This advantage becomes rather practical since all the leading consumer electronics manufacturers have released toolkits for on-device machine learning applications (*e.g.* Apple’s CoreML and Google’s TensorFlow Lite). A standalone classification algorithm, designed to operate on computationally constrained platforms, as the one presented in this paper, can run at the edge, enhancing reliability while using it in longitudinal monitoring protocols or if employed in unstructured environments and remote off-the-grid locations. By using only raw triaxial accelerometric measurements, the developed models have the potential to be highly generalizable to unspecified devices. In this perspective, authors developed one such classifier and carefully examined the trade-off between accuracy, model size and runtime complexity.

## Results

### Convolutional neural network hyperparameters optimization

**Figure 1:**
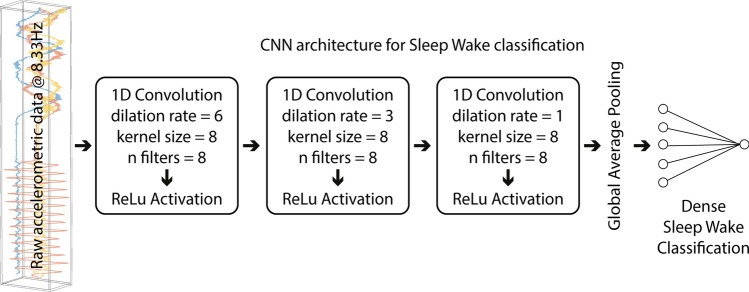
Simplified CNN architecture representation, named lightCNNA. For each layer, the layer type used and its main hyperparameters are reported.

After several iterations (see “Methods” section), the following Convolutional Neural Network (CNN) architecture (named by the authors lightCNNA, see Fig. 1) was implemented and it is constituted by the following parameters: (1) number of convolutional 1D layers: 3, (2) number of filters: 8, (3) kernel’s size: 8, (4) dilation rate first convolutional 1D layer: 6, (5) dilation rate second convolutional 1D: 3, (6) dilation rate third convolutional 1D: 1, (7) use of bias vector in convolutional layers: false, (8) use of bias terms in dense layers: false, (9) number of units dense classification layer: 16, and (10) activation function used: rectified linear unit (ReLU) for all, except the final dense layer output unit, which endowed a sigmoid activation function.

### Comparisons between PSG and equivalent lightCNNA sleep measures

**Figure 2:**
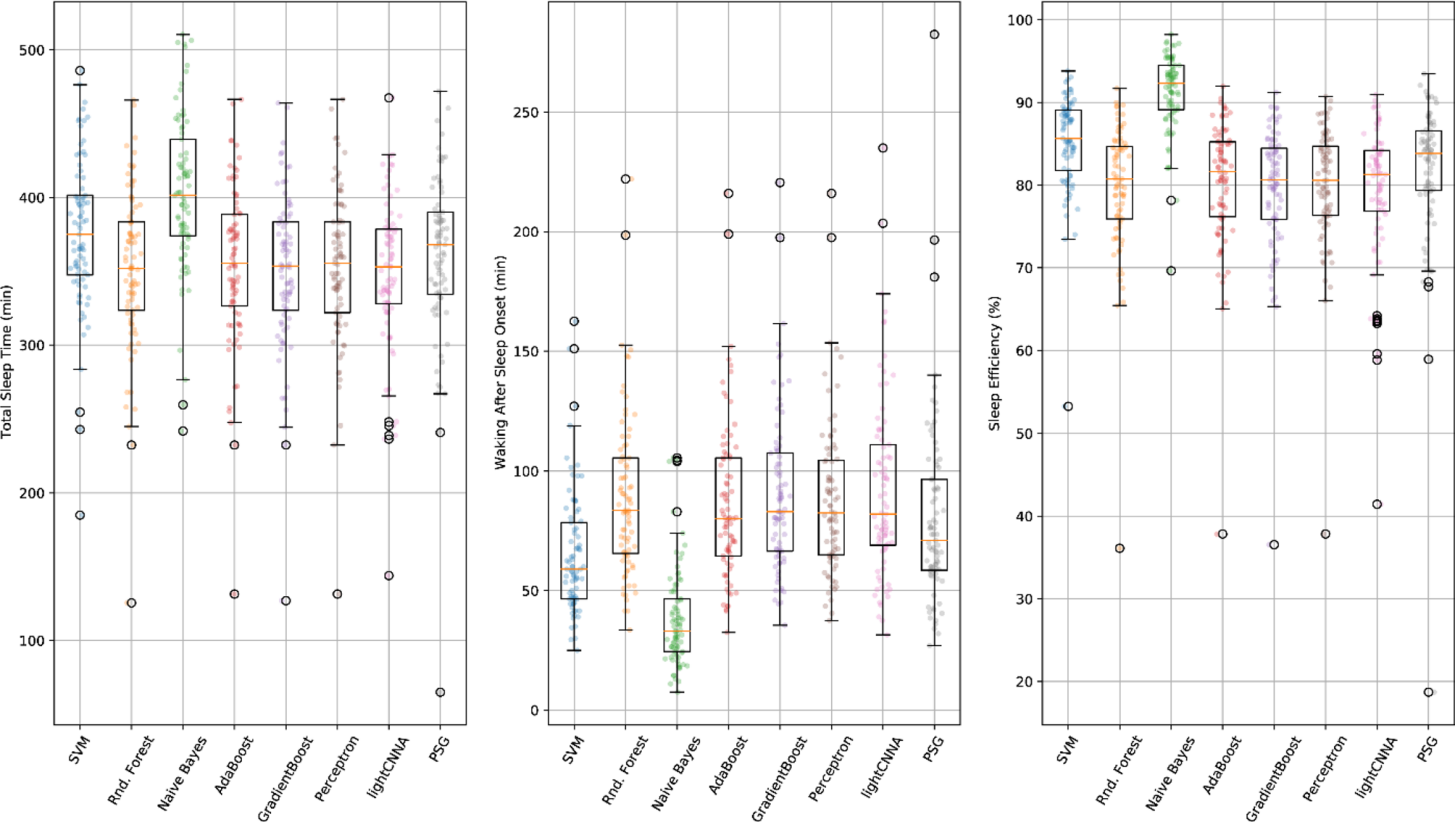
A comparison of the main sleep metrics calculated using PSG, lightCNNA and other alternative machine learning models for each subject included in the Leave One Subject Out (LOSO) validation procedure.

PSG and lightCNNA sleep measures are displayed in Table 1. Total Sleep Time (TST), Waking After Sleep Onset (WASO), and Sleep Efficiency (SE) were found to be statistically different from each other although with modest absolute differences in their mean values (PSG *minus* lightCNNA: TST 15 min, WASO -11 min, SE -2.5%, Fig. 2).

**Table 1:** Comparison of sleep metrics computed using the lightCNNA and the relative gold standard-derived values.

	PSG		lightCNNA		Statistics	
	Median ± IQR	Min–Max	Median ± IQR	Min–Max	Shapiro–Wilk p	Dunn’s p
TST (min)	368.0 ± 56.7	65 – 472	353 ± 52	144 – 467.5	0.003	< 0.05
WASO (min)	71 ± 40.5	27 – 282.5	82 ± 43.5	31.5 – 235	> 0.001	< 0.05
SE (%)	83.8 ± 7.3	18.7 – 93.5	81.3 ± 7.5	41.4 – 90.9	> 0.001	< 0.05

### Bland–Altman Plots

**Figure 3:**
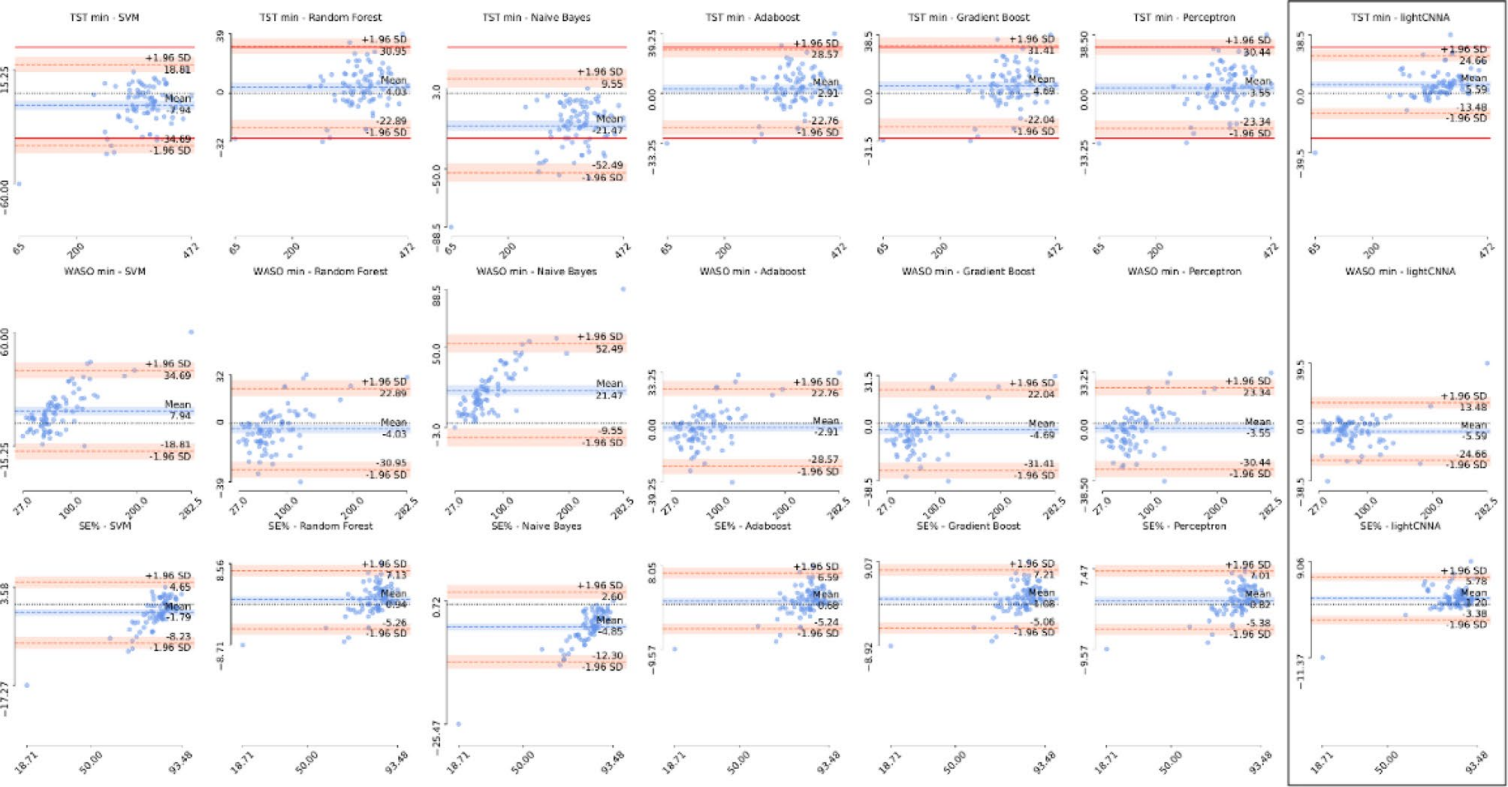
Estimation error for each sleep metric. Bland–Altman plots showing the difference in sleep metrics obtained using each of the machine learning models and the lightCNNA (highlighted by a black box) with respect to the PSG reference. The first row shows total sleep time for each model, the second waking after sleep onset, and the third sleep efficiency. Solid red lines identify the a priori acceptable limits of agreement of ± 30 min difference on TST. A dashed black line shows the zero error or perfect agreement with the PSG. Scaling is kept constant for each figure. For each axis we show the minimum and maximum values. The values reported on the y-axis are computed as PSG reference value minus the value computed by the alternative method.

Bland–Altman plots for TST, WASO, SE are reported in Fig. 3. The calculated average biases (TST 5.59 min, WASO -5.59 min, and SE% 1.20%), and a priori set clinically satisfactory limits for TST and WASO (discrepancies ≥ 30 min)^12^ are summarized in Table 1. To compute the sleep metrics, we isolated the true night using the true sleep onset and offset manually determined on the PSG scoring.

### Epoch-by-Epoch (EBE) analysis

**Figure 4:**
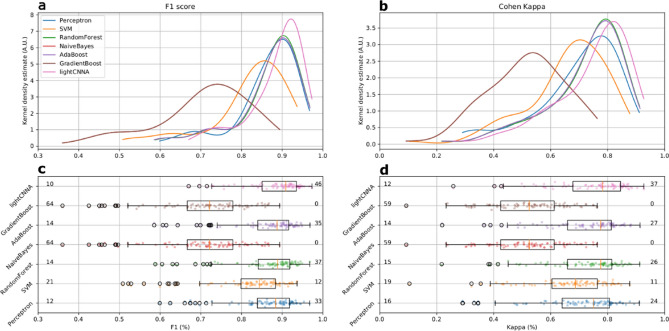
Comparison of kappa and F1 performance metrics scored by the lightCNNA model and by all other machine learning approaches. All data were computed using the LOSO approach Each point represents a subject. The number of subjects with a F1 score below 0.8 or above 0.9 is shown in panel c, at the left and right side of each boxplot. The corresponding number of subjects scoring kappa above 0.8 or below 0.6 is shown in panel d.

Overall, lightCNNA had 92.02 ± 3.11% specificity (ability to detect wake), 89.23 ± 3.46% sensitivity (ability to detect sleep), 89.32 ± 3.36% concordance, and 90.88 ± 3.04% F1 score, relative to PSG (see Table 2 and Fig. 4).

**Table 2:** Comparison of performance metrics scored by the lightCNNA model and other machine learning approaches. An * denotes the presence of a statistically significant difference between lightCNNA and other algorithms.

	Kappa	F1	Concordance	Specificity	Sensitivity
Perceptron	0.751	0.884	0.876	0.899	0.872
SVM	0.687*	0.844*	0.843*	0.972*	0.742*
RandomForest	0.776	0.889	0.888	0.948	0.842*
NaiveBayes	0.524*	0.721*	0.759*	0.983*	0.573*
AdaBoost	0.775	0.887	0.887	0.948	0.842*
GradientBoost	0.524*	0.721*	0.759*	0.983*	0.573*
**lightCNNA**	**0.782**	**0.909**	**0.893**	0.920	0.892*

The overall Cohen’s kappa coefficient (CKC) for the lightCNNA, as compared to PSG, was 0.78 ± 0.07. All metrics are presented as median ± mean amplitude deviation. Table 2 reports the EBE performance of each machine learning algorithm implemented.

### Optimization of lightCNNA output binarization

**Figure 5:**
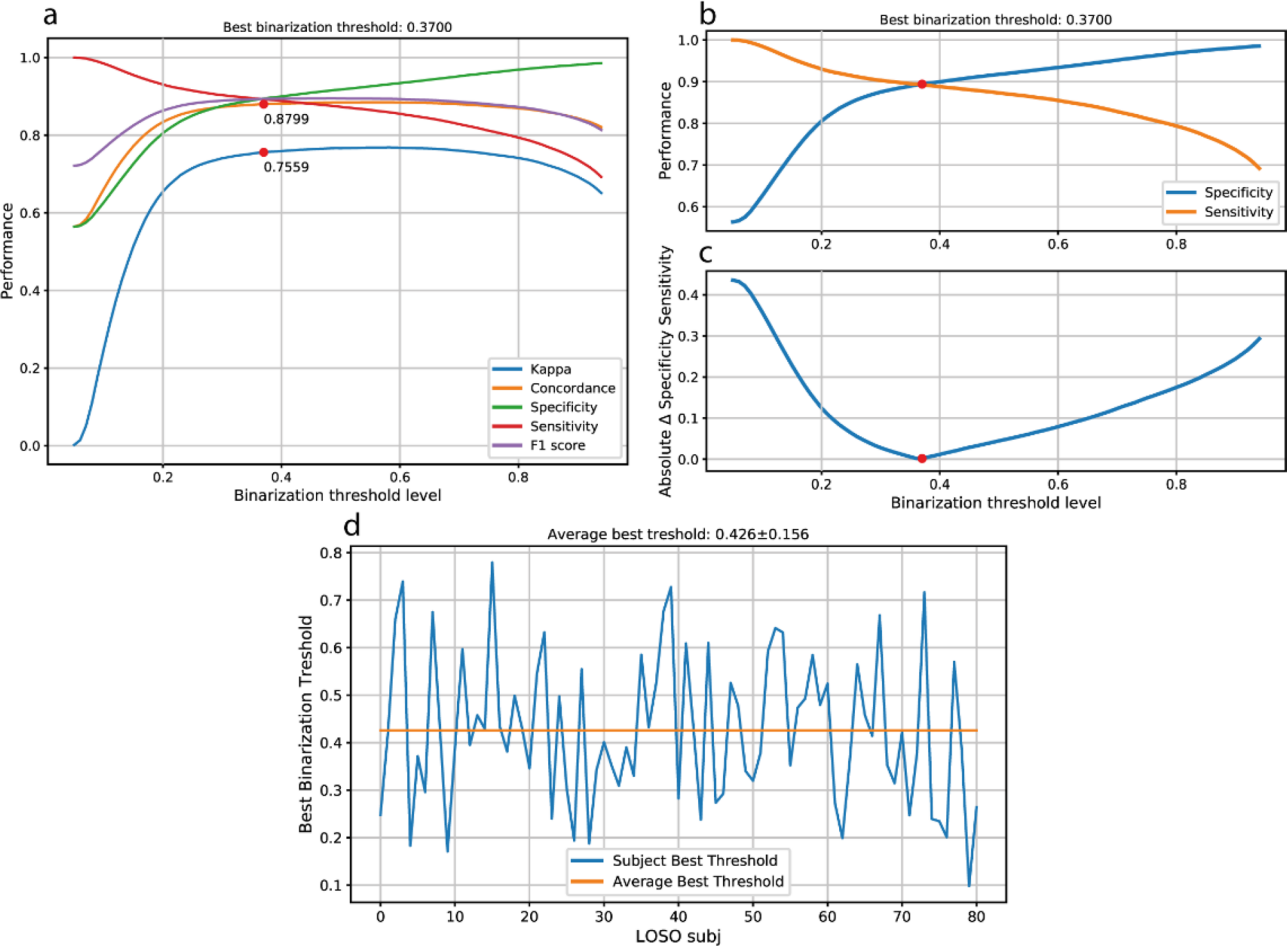
Optimization of binarization threshold of lightCNNA output. (**a**) shows the effect on models performances of the variation of the binarization threshold. (**b**) highlights the point in which the classifier maximizes concordance, by showing the minimum absolute difference between specificity and sensitivity (**c**). (**d**) shows the value of the best threshold estimated for each subject during the LOSO validation.

A binarization threshold of 0.370 was optimized on the hold-out training set (Fig. 5a,b,c). Using the data gathered trough the leave one subject out (LOSO) validation scheme, we computed a threshold value for each subject (see “Methods”section, n = 81). By averaging across subjects, a further binarization threshold equal to 0.426 (Fig. 5d) was obtained.

**Figure 6:**
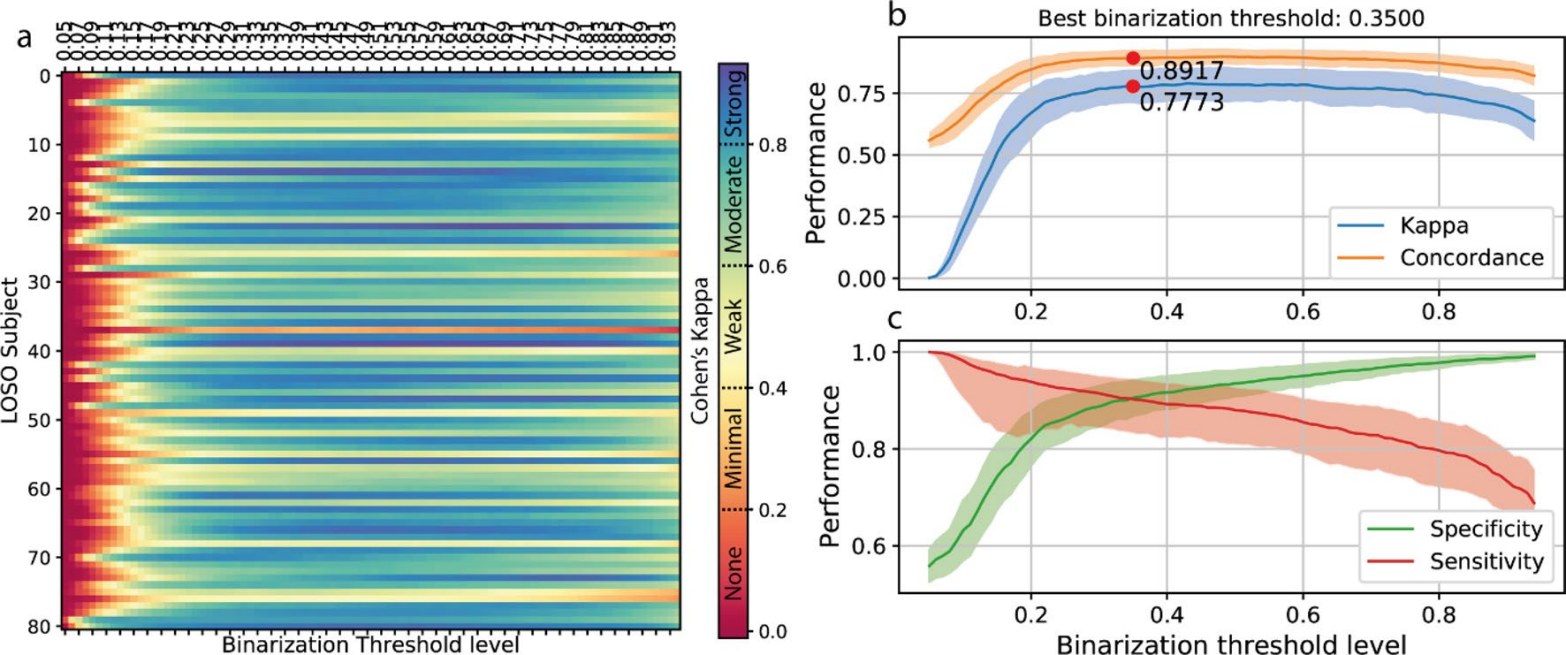
Variation of binarization threshold on single LOSO subject data. (**a**) Effect of variation of the binarization threshold level on CKC for each LOSO subject. Performance achieved using a specific binarization treshold: in (b) CKC and concordance, in (c) specificity and sensitivity; solid lines represent median values, the shaded area shows the corresponding mean amplitude deviance.

Finally, varying the binarization threshold on each single LOSO subject resulted in an optimal threshold of 0.350 (Fig. 6). The overall variation in models performance was small (average absolute variation of CKC 0.013 ± 0.011) across threshold estimation techniques, hence we adopted the personalized threshold (one for each subject, n = 81) to measure models performance at its optimal working point.

## Discussion

Currently available algorithms used to automatically detect and score sleep, based on actigraphic data, exhibit a common limitation in the relatively low level of classification reliability (average ± standard deviation CKC of 0.45 ± 0.15, n = 4, see Table 2 and Supplementary Table 1) and specificity achieved (average ± standard deviation of 60.7 ± 22.0%, n = 21, minimum of 32.9%^11^, see Table 2 and Supplementary Table 1). This significantly limits the application of actigraphy in the diagnosis of sleep disorders^13^, and more specifically of chronic insomnia, as specificity reflects the capability of detecting awakenings. In an attempt to overcome this limitation, we implemented a machine-learning approach obtaining good results in terms of specificity (average ± standard deviation of 89.33 ± 7.85%, median ± mean amplitude deviance of 89.23 ± 3.46%), at the expense of a slight reduction of sensitivity (average ± standard deviation of 87.66 ± 6.28%, median ± mean amplitude deviance of 92.02 ± 3.11%), in comparison with commonly used algorithms (Table 2 and Supplementary Table 1). Moreover, the lightCNNA exhibits high reliability in the binary classification of sleep and waking, achieving a CKC of 0.78 ± 0.07 (median ± mean amplitude deviance, or 0.75 ± 0.13 average ± standard deviation).

The lightCNNA showed a good agreement with PSG in the whole night estimation of TST, WASO and SE in this heterogenous group of adults, with 85.19% of the participants lying within the a priori-set clinically satisfactory ranges for TST and WASO (≤ 30 min difference)^12^. The Bland–Altman plot limits of agreement for TST, WASO and SE of the current study were significantly higher as compared to both medical-grade^14^ and consumer-grade actigraphs^15–17^ (Supplementary Table 1 summarizes the metrics for a sample of published algorithms). The lightCNNA did not show clinically relevant, systematic TST, WASO and SE overestimation, underestimation or magnitude related trends (Fig. 3).

Overall the lightCNNA model proved to be better than all other algorithms in every comparative metrics, except for specificity. Regarding specificity (see Table 2), this value was higher for other algorithms (Naïve Bayes and Gradient Boost) at the expense of sensitivity. When considering the most compelling comparison metrics, such as Kappa and F1, the lightCNNA model showed a more robust performance (see Fig. 4) by scoring: the highest number of test subject with a F1 score above 0.9 and the highest number of test subject with kappa values above 0.8, while the lowest number of test subject with a F1 score below 0.8 and the lowest number of test subject with kappa values below 0.6. An important issue to highlight is the epoch duration, *i.e.* the temporal resolution for the estimation of epoch-by-epoch performance comparison. In this paper, we used a 30 s time window while others (Table 3) reduced the temporal resolution to 60 s. Manipulating the time resolution also imposes to modify the ground truth reference time series, as PSG scoring is done on 30 s epochs. This procedure may add noise into the ground truth labels as custom re-scoring rules should be created to solve ambiguities creating new epochs from non-homogeneous source labels. As an example, Aktaruzzaman et al.^18^ considered epochs containing both NREM sleep N1 sleep and waking as waking, Sadeh et al.^19^ scored as wake any mixed epochs, and Paquet et al.^12^ scored PSG data on 20 s epochs and re-scored the signal to a 60 s resolution using a majority criterion. The result of these approaches may alter the ability of machine learning methods to correctly identify brief transitions between behavioral states due to injection of noise in the training ground truth data. Hindering a method ability to detect brief awakenings during night time is relevant, as the nature of these events is limited to a few epochs but may account for a significant fraction of clinically relevant infra-sleep waking over the night, and ultimately improves the specificity achieved by the scoring method.

**Table 3:** Comparison of methodologically relevant parameters of other algorithms reported in literature.

Reference	Epoch duration (s)	Population size	Avg. subject recording duration (minutes)
Aktaruzzaman et al. ^18^	390	18	–
Blood et al.^20^	–	9	–
Cole et al.^21^	60	41	443.7 ± 61.5
de Souza et al.^22^	60	21	–
Domingues et al.^23^	30	29	–
Farabi et al.^24^	30	27	–
Haghayegh et al.^25^	30	40	–
Hedner et al. ^26^	30	228	–
Jean-Louis et al. ^27^	30	5	480
Khademi et al.^28^	30	54	–
Kosmadopoulos et al.^29^	30	22	–
Kushida et al.^30^	30	100	–
Li et al.^31^	400	10	–
Lichstein et al.^32^	30	57	–
Long et al.^33^	30	25	396 ± 54
Marino et al.^11^	30	77	–
Palotti et al.^10^	30	1817	–
Paquet et al.^12^	60	15	–
Pollak et al.^34^	30	28	~ 10,000
Roberts et al.^35^	30	8	–
Sadeh et al.^36^	60	36	–
Sivertsen et al.^37^	30	34	–
lightCNNA	30	81	858 ± 132

A strength of the proposed classification model is the ability to process raw accelerometric data, reducing the complexity and the computational costs of the method, while maximizing its generalizability. The use of CNNs also allows to avoid the time-consuming and inherently suboptimal feature engineering process as the model can autonomously learn relevant features during training. CNNs are also easily extensible to new sources of information (*e.g.* photoplethysmography, acoustic, anamnestic) providing possibilities to expand the applicability to a wide array of data types and tasks. As an example, the use of raw photoplethysmographic data may enable the introduction of breathing and circulatory variables. Breathing patterns variations are particular relevant in the diagnosis of breathing-related sleep disorders (and may be collected on the wrist^38,39^), but are also associated to the physiological sleep onset process^40^, and might be used to compensate for the systematic differences in sleep onset estimation between PSG and actigraphy.

Methodologically, a significant advantage of the implemented approach consists of the following strengths: (1) the data used in our work come from relatively long monitoring windows (about 14 h on average), including a representative balanced sample of both spontaneous waking, as well as sleep, and (2) the concurrent portable PSG and actigraphic recordings allowed for a rather naturalistic approach, as the subjects spent their monitoring time in a familiar, comfortable and usual environment and not in the hospital or laboratory. Moreover, as the source of information feeding this algorithm is only a triaxial accelerometer, the approach is easily generalizable to a vast number of actigraphic devices coming from both the medical-grade and the wearable consumer realm. This characteristic helps to make the lightCNNA model more generalizable than those developed using data recorded by conventional actigraphs. In fact, those model cannot be easily used across brands as usually each actigraph encodes movement into slightly different “actigraphic counts”, ultimately limiting generalizability. This type of data is not the raw acceleration recorded by the embedded sensors but, usually, is an integral of the acceleration over a certain period of time or other proprietary conversion of the raw acceleration. This approach is largely inherited by the previous generation of actigraphic devices that had low computing power and a small embedded memory. Several of the producers of already widespread consumer devices (*e.g.* Apple, Fitbit, Garmin, etc.) provide a simplified way to log and/or stream data from the accelerometer embedded in their products. This is beneficial as it greatly widens the range of compatible devices and simplifies the technical complexities of building a custom hardware-software infrastructure for data collection purposes. Using already available hardware with simplified programming interfaces is also beneficial as it enables the integration of additional modules that can collect user inputs useful to track a variety of additional variables of interest, *e.g.* therapy compliance, getting in–out of bed, caffeine consumption, anxiety rating, subject response speed, life events. The widespread possibility of recording accelerometric data from simple-inexpensive devices also enables the possibility to study sleep on vast population outside the laboratory, in an unobtrusive but reliable way for extended periods of time, beyond the PSG applicability.

The described approach allows the processing of the data entirely at the edge, using simple computing devices. This characteristic enhances the security of personal sensitive data as the approach is intrinsically safe avoiding any exchange of data over public or proprietary network and computing infrastructures. Moreover, the low sampling rate that can be used to gather data to feed the lightCNNA model simplifies power management of the device running the data collection and the inference at the edge. In fact, lowering sensors sampling rate reduces the power usage and allows the device to “*sleep*” when not actively used (*i.e.* powering only a subset of peripherals-sensors interfaces or drastically reducing clock speed).

Moreover, future developments of the lightCNNA model might go in the direction of improving performance trough personalization, as seen in other recent approaches^28^. If on one side it is not easily conceivable to run a training procedure on device, on the other side some personalized descriptors (*e.g.* age, overall sleep wake cycle architecture, sleep regularity) might improve the output of the model. However, this would require a large person-specific dataset, paired with a complex technical implementation enabling the fine-tuning of a general model such as lightCNNA. A possible novel application of our lightCNNA could be developed in conjunction with bio-mathematical modeling of attentional levels^41,42^ to develop automatic algorithms able to estimate and possibly mitigate the exposure to sleep deprivation, a raising concern of our society^43^. This tool may also be investigated as a way to reduce accident risk due to the effects of sleep deprivation^44,45^, *e.g.* during safe sensitive and around the clock tasks, such as surgery.

A limitation of lightCNNA is that it cannot distinguish sleep stages. Possible improvements derive from: (1) the use additional physiological signals (*e.g.* photoplethysmography)^46–48^ to overcome, at least in part, the impossibility to detect the sleep stages and further enhance the robustness and accuracy of the model including exogenous inputs to the model mapping anamnestic and behavioral information, and (2) the possibility to synthetize high fidelity physiological signals, using custom-built generative adversarial models to mitigate the paucity of data or its unbalancing.

## Methods

### Participants

81 subjects were recruited in this study (average age of 23.4 ± 5.2 yrs). All subjects were enrolled within the Pisa University Hospital, Pisa, Italy. The sample included both healthy as well as subjects undergoing a diagnostic exam for sleep disturbances. Each participant was equipped with a portable PSG system (Morpheus, Micromed SpA, Mogliano Veneto, Italy) and an open-source actigraph (Axivity AX3, Axivity Ltd., Newcastle upon Tyne, United Kingdom) placed on the wrist of the non-dominant hand. The study was carried on in accordance with relevant national and regional regulations and following the principles detailed in the Declaration of Helsinki. The local ethical committee (Azienda Ospedaliero Universitaria Pisana, Ethical comitee Area Vasta Nord Ovest, Approval number 987 Protocol number 13711) approved the experimental protocol and subjects filled a written informed consent before the beginning of the study. Subjects were monitored overnight and spent the night at home in their usual sleeping environment. The mean acquisition duration was 14.3 ± 2.2 h. The Axivity AX3 is an open-source device equipped with a triaxial accelerometer (ADLX345) and 512 MB of on NAND flash memory. PSG data were sampled at 512 Hz for the 12 EEG derivations (F3, F4, C3, C4, T3, T4, P3, P4, T5, T6, O1, O2, P, ground in Cz, reference in Fz), 1 EKG (bipolar derivation placed symmetrically around the sternum within the 3^rd^ and 4^th^ ribs), 2 EOG (left and right vertical), and 2 EMG derivations (electrodes placed on the chin over the suprahyoid muscles). Raw triaxial acceleration was recorded at a mean frequency of 99.7 ± 2.3 Hz with a 10bits resolution. PSG data were exported in EDF + format, imported in Alice (Koninklijke Philips N.V., Amsterdam, The Netherlands), and visually scored based on 30 s epochs by an expert technician following AASM criteria^1^.

### Convolutional neural network

The sequential nature of actigraphy data motivates the use of Convolutional Neural Networks (CNNs). Various network architectures were implemented and tested to achieve the maximum accuracy, while keeping the computational cost as low as possible. All models were developed using open source software: Python 3.6 (Python Software Foundation), Keras-GPU 2.2.4^49,^ and TensorFlow-GPU 1.13.1^50^. Training was accomplished using a Nvidia GeForce GTX 1080 Ti GPU (Nvidia Corp., Santa Clara, California, USA). All plots were created using matplotlib^51^ and GIMP 2.10.22^51^. The following parameters were systematically investigated, and the best performance was selected based on the highest CKC and concordance between the actigraphic-based binary scoring and the visual EEG-based gold standard scoring, within the test set. Models’ hyperparameters tuned are: (1) number of convolutional 1D layers, (2) number of filters, (3) kernel’s size, (4) dilation rate of each convolutional layer, (5) use of bias vector in convolutional layers, (6) use of bias terms in dense layers, (7) number of units dense classification layer, and (8) activation functions.

For equal CKC performance, the architecture with the lowest Floating-Point Operations (FLOPs) was selected. The resulting architecture (Fig. 1), named lightCNNA, counts a total of 1361 parameters (all trainable). The overall computational cost was estimated to be 2727 FLOPs.

The lightCNNA model can be converted to a format suitable to be embedded in iOS or Android applications using the TensorFlow Lite converter. Furthermore, the model might be deployed on targets without an operating system by converting the TFLite model, obtained using the aforementioned converter, to a C array format optimized for suitable targets.

### Dilated convolutions and the role of context

Increasing the kernel size improves performance at the expense of computational complexity. A larger kernel can process more information from its input at the same time which, for example, can be beneficial for the detection of sleep/wake transitions. From this point of view, a larger kernel can serve the same purpose as the hidden states in recursive models. In this study, authors take advantage of dilated (a trous) convolutional layers to increase the receptive field of the network with a limited computational load. While in standard convolutional layers the kernel is directly convolved over the input, in dilated convolutions the kernel is resampled over a larger area, effectively adding “*holes*” to the convolution operation. The kernel resampling rate is controlled by the dilation rate. Dilated convolutions are a powerful method to grow the receptive field without increasing the kernel size (and thus the computational load).

### Optimization of models output binarization

The raw output of the lightCNNA is a floating-point number comprised between zero and one representing the probability of a certain epoch of being labelled as wake. An optimal threshold for a balanced binary classifier can be set as the point where the true positive rate is highest, for the lowest number of false positive misclassifications. We estimated the optimal binarization threshold using the standard hold-out test set data, already used for model hyperparameter optimization (Fig. 5a,b,c). We computed the performance metrics achieved by the model, while modifying the binarization threshold level between 0.05 and 0.95 with an increment of 0.01. Additionally, while computing lightCNNA performance within the LOSO validation approach, each subject test data was binarized using the optimal threshold estimated using the very same data of the subject itself (Fig. 5d). Lastly, for each LOSO iteration we binarized each subject data with the thresholds array as defined for the binarization of the hold-out dataset (Fig. 6). Then, for each threshold level, we averaged CKC, concordance, specificity and sensitivity across subjects. Using the averaged data, we identified the last binarization threshold value.

### Accelerometer sampling rate

Authors simulated lower sampling rates by decimating the accelerometer time series without the use of filters or interpolation techniques to better approximate slower sampling accelerometers. Results in Supplementary Fig. 1 show that data sampled at 8.3 Hz performed with the highest CKC (0.72). Moreover, we chose not to use higher sampling frequencies as the frequency of most voluntary human movements spans from 0.6 to 8Hz^52,53^ and rarely (essentially limited to young subjects^54^) exceeds 4Hz^55^.

### Training regime

CNNs operate on fixed-length sequences. However, the duration of the actigraphy recordings vary from patient to patient and from night to night. For this reason, we partitioned the training time series into fixed-length chunks and shuffled them, to reduce bias, before each training epoch. The straightforward approach is to pair each PSG-labeled 30 s epoch with its corresponding accelerometer samples. However, this choice is overly restrictive, as it does not provide the network with enough context around the labelled epoch to classify it correctly. Instead, we provided a larger context window around the labeled epoch. The length of this window influences both classification accuracy and computational complexity. Each sequence of input data is built including data from the previous 30 s and to the subsequent 30 s. The applied optimizer is Adam^56^ with L2 weight normalization and the following initial parameters, *i.e.* (1) initial learning rate: 0.001, (2) beta1:0.9, and (3) beta2: 0.999.

### Data normalization

We applied a min–max normalization to raw accelerometric data to improve robustness. Data were fit in a range comprised between -1 and 1. To enhance reproducibility, we used the minmax scaler function of the sklearn preprocessing package v0.21.3 for Python 3.6.

### Synchronization of actigraphy and Polysomnography data

To ensure a reliable alignment between actigraphy and polysomnography, we performed a synchronization of the internal clocks of both the Axivity AX3 actigraph (Axivity Ltd., Newcastle upon Tyne, United Kingdom) and the Micromed PSG holter (Micromed SpA, Mogliano Veneto, Italy). Both internal clocks were updated and synced each time a new recording session was started, at the beginning of an experimental session and also at the end for the PSG data. The reference clock used to synchronize the instruments was the one of the computer used to launch the recording session of both devices. As an additional control, the PC clock was automatically kept in synchronization with an external atomic clock (Istituto Nazionale di Ricerca Metrologica, server address: ntp1.inrim.it, supported protocols: Network Time Protocol RCF-5905), using a background NTP server that updated and re-synced the local PC clock before each recording. After the experiment, each PSG start and stop timestamps were read from the header of the EDF + file storing the PSG data. The start and stop timestamps were then used to find the closest timestamps in the actigraphy raw data (non-decimated) series. The actigraph recorded a millisecond resolution time stamp for each sample acquired. As the PSG timestamps were stored using a precision of a single second, the maximum synchronization error is ± 2 s considering a same sign error for both the starting and ending of the recording.

### Performance metrics and statistical analysis

Since this study is focused on computational complexity and on-device inference, we report results in terms of per-epoch classification accuracy of the lightCNNA. To enable direct comparisons with all implemented machine learning models and with the available literature in the field, we report the following epoch-by-epoch metrics:

1 $$\mathrm{Accuracy}= \frac{\mathrm{TP}}{\mathrm{TP}+\mathrm{TN}+\mathrm{FP}+\mathrm{FN}}$$

2 $$\mathrm{Sensitivity}= \frac{\mathrm{TP}}{\mathrm{TP}+\mathrm{FN}}$$

3 $$\mathrm{Specificity}= \frac{\mathrm{TN}}{\mathrm{TN}+\mathrm{FP}}$$

4 $$\mathrm{F}1\mathrm{ score}=(\frac{2}{{\mathrm{sensitivity}}^{-1}+{\mathrm{specificity}}^{-1}})$$

5 $$\mathrm{Kappa}= 1-\frac{\mathrm{accuracy}- {\mathrm{p}}_{\mathrm{e}}}{1- {\mathrm{p}}_{\mathrm{e}}}$$

6 $$Precision= \frac{TP}{TP+FP}$$

7 $$Recall= \frac{TP}{TP+FN}$$

In Eq. (5), the term p_e_ represents the expected chance agreement (see ^57^ for details), whereas TP and TN represent true positive and true negative samples.

In the comparison of EBE performance metrics reported above, for each proposed algorithm, we employed a Shapiro–Wilk test to probe data normality. Since data were not normally distributed, we opted for a Kruskall-Wallis test (one way) followed by multiple comparison test administered using the Dunn’s correction method.

Moreover, we implemented the Receiver Operating Characteristic (ROC) curves and their Area Under Curve (AUC) (Supplementary Fig. 2a). Since ROC curves and AUC can be a misleading goodness-of-fit metric for classifiers dealing with unbalanced datasets^58,59^, we also include Precision-Recall Plots (Supplementary Fig. 2b). Sleep metrics were compared using a one way Friedman repeated measures analysis of variance on ranks followed by multiple comparison procedure using the Dunn’s method, after checking for normality (Shapiro–Wilk test). When appropriate, we reported median values accompanied by their median absolute deviation. As a measure of computational complexity, we report the total number of FLOPs for each model configuration. FLOPs can be computed directly through the analysis of the network architecture (*e.g.*, depth and convolutional kernel size, etc.) and are an acceptable proxy for power consumption of the computing device. To enhance reliability and reproducibility of the calculation of this metric, we used the built-in TensorFlow 1.13.1 model profiler.

Since hyperparameter search space is relatively wide, only during the preliminary phase of hyperparameter optimization we evaluated our models using the aforementioned metrics calculated on a hold-out validation set, *i.e.* a randomly picked 20% of all the available data. After this preliminary phase, a comprehensive LOSO validation scheme was adopted to calculate final performance metrics of each model. If not stated otherwise, we only report the results calculated using the LOSO approach.

### Alternative machine learning models

We implemented an array of seven machine learning models alternative to lightCNNA. All models are features based. Then, for each 30 s epoch, we computed a vector containing the median, standard deviation, minimum and maximum value of the accelerometric input data for each of the three axis of the inertial sensor. Hence a total of 12 features were fed to each classifier. Implemented machine learning classifiers were: (1) Linear Support Vector Machine, (2) Random Forest, (3) Naïve Bayes, (4) AdaBoost, (5) Gradient Boost, and (6) a swallow neural network or perceptron. Models were implemented using the open source software modules, *i.e.* SciKit-learn 0.22.2^60^, Keras 2.2.4, and TensorFlow 1.12.0.

## Supplementary Information

Supplementary Information.

## Data Availability

Anonymized data, used with permission for the current study, are available upon request, according to data protection policies defined by the Ethical Committee approval.
