# Supplementary material for: Efficient embedded sleep wake classification for open-source actigraphy
Source: Sci Rep. 2021 Jan 11;11:345. doi: 10.1038/s41598-020-79294-y (PMC7801620; doi:10.1038/s41598-020-79294-y)
Supplement: Supplementary file 1 — Supplementary Information. [file 41598_2020_79294_MOESM1_ESM.pdf]

Supplementary Material

-

Efficient embedded sleep wake classification for open-source actigraphy

T. Banfi*[1,2,3], N. Valigi[3], M. di Galante[3,4], P. d'Ascanio[5], G. Ciuti[1,2] and U. Faraguna[3,4,5]

** Corresponding author*

Contact information:

Tommaso Banfi

Email: tommaso.banfi@santannapisa.it

Address: The BioRobotics Institute, Viale Rinaldo Piaggio 34, Pontedera 56025, Italy.

Phone: +39 050 883057

***Affiliations*:**

[1]The BioRobotics Institute, Scuola Superiore Sant'Anna, Pisa, Italy. [2]Scuola Superiore Sant'Anna, Department of Excellence in Robotics & AI, Pisa, Italy. [3]sleepActa S.r.l., Pontedera, Italy. [4]IRCCS Stella Maris, Department of Developmental Neuroscience, Pisa, Italy. [5]Department of Translational Medicine and of New Technologies in Medicine and Surgery, University of Pisa, Pisa, Italy.

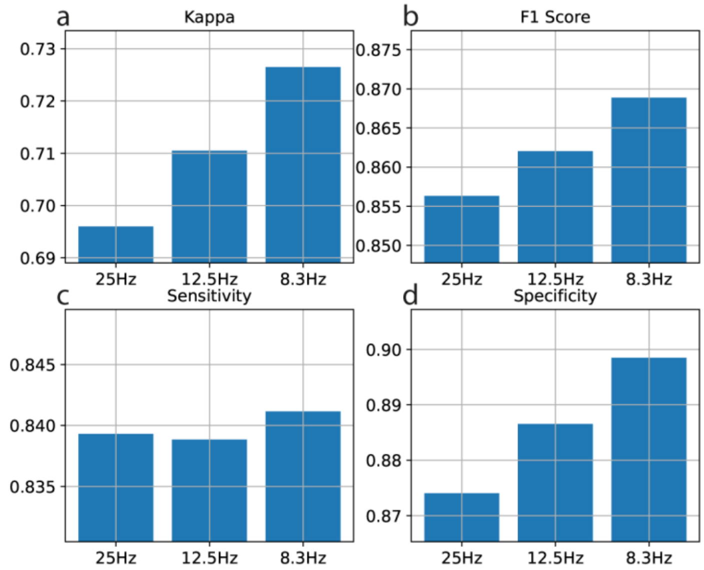

Supplementary Figure 1 - Effect of decimation of the input series to various simulated sampling rate. (a) Shows

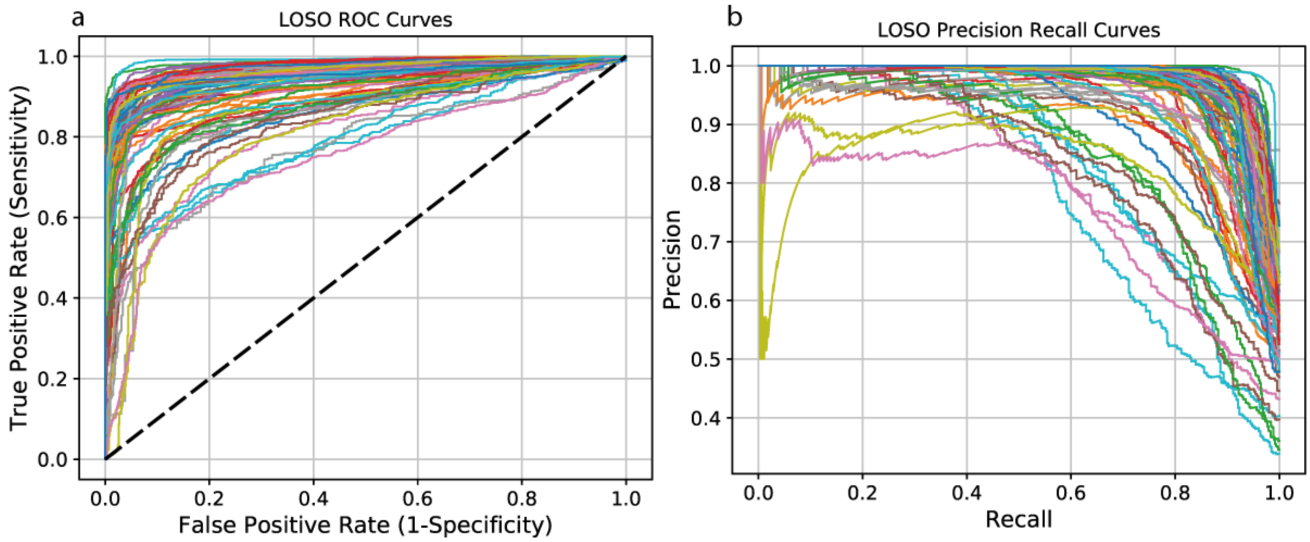

Supplementary Figure 2 - Precision Recall and ROC Curves. Curves Calculated using the LOSO approach. (a) Shows the ROC curves for each subject compared to a random classifier (dashed black line). Instead (b) shows the precision recall curves.

Supplementary Table 1 - Comparison of performance achieved by several actigraphic algorithms reported in literature.

Reference	Accuracy %	Specificity %	Sensitivity %	Cohen's kappa coefficient (CKC)
(Aktaruzzaman *et al.* 2017)[18]	78±8	89±5	33±16	0.31±0.17
(Blood *et al.* 1997)[40]	82±2	35±9	95±1	-
(Cole *et al.* 1992)[41]	88	64	95	-
(de Souza *et al.* 2003)[42]	91	34	99	-
(Domingues *et al. 2014*)[43]	75.5	73.5	73.8	-
(Farabi *et al.* 2017)[44]	78	-	-	0.25
(Haghayegh *et al.* 2019)[45]	86.1	48.6	97.0	51.3
(Hedner *et al.* 2004)[46]	86	69	89	-
(Jean-Louis *et al.* 2001)[47]	91.3	40.6	94.8	-
(Khademi *et al.* 2019)[48]	86	99	26	-
(Kosmadopoulos *et al.* 2014)[49]	87.7±7.6	37.7±14.5	95.8±2.7	35±12
(Kushida *et al.* 2001)[50]	80	45	90	-
(Li *et al.* 2018)[51]	88	91	82	0.74
(Lichstein *et al.* 2006)[52]	-	-	-	-
(Long *et al.* 2017)[53]	84	92	60	0.55
(Marino *et al.* 2013)[11]	86.3	32.9	96.5	-
(Palotti *et al.* 2019)[10]	87.7±2.3	86.6±2.9	90.1±4.1	-
(Paquet *et al.* 2007)[12]	90	54	95	-
(Pollak *et al.* 2001)[54]	78.2	62.2	96.4	-
(Roberts *et al.* 2020)[55]	88±5.4	64.7±16.3	91.2±6.4	48.7±14.6
(Sadeh *et al.* 1994)[56]	92	75	96	-
(Sivertsen *et al.* 2006)[57]	83	36	95	-
(Slater et al. 2015)[58]	84	46	90	-

**LightCNNA (mean ± SD)**	**88.43±6.23**	**89.33±7.85**	**87.66±6.28**	**0.75±0.13**
**LightCNNA (median±mean amplitude deviance)**	**89.32±3.36**	**92.02±3.11**	**89.23±3.46**	**0.78±0.07**
